# Youth sport participation and physical activity in rural communities

**DOI:** 10.1186/s13690-021-00570-y

**Published:** 2021-04-08

**Authors:** Debra K. Kellstedt, Michaela A. Schenkelberg, Ann M. Essay, Mary J. Von Seggern, Richard R. Rosenkranz, Gregory J. Welk, Robin High, David A. Dzewaltowski

**Affiliations:** 1 College of Public Health, University of Nebraska Medical Center, Omaha, NE USA; 2 School of Health and Kinesiology, University of Nebraska at Omaha, Omaha, NE USA; 3 College of Health & Human Sciences, Kansas State University, Manhattan, KS USA; 4 College of Human Sciences, Iowa State University, Ames, IA USA

**Keywords:** Physical activity, Rural, Youth sport, Health equity

## Abstract

**Background:**

Physical activity, a high-frequency health behavior, varies by where children live, learn, and play. Children accumulate physical activity in adult-led in-school and out-of-school settings. Youth sport is a potential setting for physical activity, but there are differences in youth sport participation based on age, sex, and socioeconomic status. There is a gap in understanding demographic influences on youth sport participation and how these factors interact to influence physical activity. This study examines influences of grade, sex, and family income on youth sport participation and these factors and youth sport participation on moderate-to-vigorous physical activity of children in rural communities.

**Methods:**

Children (*n* = 418 3rd–6th graders) living in two rural communities completed the online Youth Activity Profile as part of Wellscapes, a type 3 hybrid implementation-effectiveness community randomized trial. Mixed models with community as a random effect examined main effects and interactions of grade, sex, and family income on youth sport participation and these factors and youth sport participation on moderate-to-vigorous physical activity.

**Results:**

About 80% of children engaged in youth sport, and full-pay lunch students were almost four times more likely to have youth sport participation than students with free/reduced lunch (OR = 3.91, 95% CI = 1.95, 7.8). Females and 6th graders (*p* < 0.05) had lower physical activity than comparison groups. Males with higher family income had greater physical activity; females with higher family income had less physical activity. For 6th graders, high family income had less effect on physical activity than similar 3rd-5th graders (*p* < 0.01).

**Conclusions:**

While a fairly high percentage of children participate in youth sports, there are disparities in rural communities on youth sport participation and physical activity outcomes based on age, sex, and family income.

## Background

Physical activity (PA), a high-frequency health behavior, varies by where children live, learn, and play [1]. Establishing healthy behaviors early in life can reduce the risk of certain types of cancers and other chronic conditions that can develop over the life course [2, 3]. The current Centers for Disease Control and Prevention guidelines indicate that children and youth ages 5–17 years should accumulate at least 60 min of moderate to vigorous physical activity (MVPA) every day [4], but in recent estimates, only 21% of children in the U.S. are meeting those guidelines [5]. Physical inactivity and obesity are greater problems in rural communities than in urban or suburban environments [1, 6–8], and children’s PA behaviors are influenced by the social and physical community environments that surround them [9–12]. In the past, children’s PA occurred in free range settings while walking or biking to school and while playing on neighborhood streets and in parks [13]. In contemporary community social structures, children’s PA occurs more frequently in adult-led in-school and out-of-school settings, like physical education (PE) classes or youth sport [13, 14]. PE is a teacher-led class in schools that provides curricula aimed at improving physical skills, knowledge, and behaviors [15]. A 2008 cross-sectional study found rural and urban differences in these settings for children’s PA with more activity for rural children than urban at lunchtime and after school but less during PE classes [1]. A 2016 systematic review of PA-related policy and environmental strategies to prevent obesity in rural communities found that one of the most common intervention strategies to prevent obesity was to increase opportunities for out-of-school PA [14]. As noted in the study, an example of a policy employing this strategy would be to share school facilities, like outdoor fields, for public use before or after school.

For over 50 years, playing sports has been reported as a favorite activity of children [16, 17], and youth sport holds potential as a setting for health promotion [14, 18]. In the past, youth sport, through youth development programs, was seen as a social solution for keeping children occupied in positive activity and less likely to engage in problem behaviors [19, 20]. More recently, youth sport has been promoted for its physical, psychological, and even intellectual benefits [21, 22]. A 2013 systematic review found considerable evidence for improved psychological and social health from youth sport participation over other forms of leisure-time PA [23]. A 2007 study of boys aged 6–12 years found that youth sport participation contributed to PA benefits with 23% of total daily minutes of MVPA—a higher percentage than either PE or recess [24]. A 2006 study of adolescent girls demonstrated that sport participation contributed to girls’ vigorous PA in late adolescence, and that having more years of sports participation was associated with more PA [25]. A more recent study conducted in Europe found that participation in sports doubled the odds of meeting PA guidelines among 7–12-year-olds [26]. In addition to immediate benefits, youth sport participation can lead to more PA in adulthood [27, 28].

Youth sport holds promise for impacting population health, because this setting has the potential to reach a large number of children [29]. In the U.S., over 60 million children participate in youth sport [30]. But with youth sport programming moving away from the school setting and resulting in increased costs and focus on competition, youth sport may not be accessible to all [30, 31]. In Australia, a decline in youth sport participation is attributed to limited access due to costs of uniforms and registration [13]. According to the National Survey of Children’s Health, 64% of U.S. children aged 6–17 years are participating in sports, but this number varies by age, sex, and family income [32]. Past research has shown a decline in sports participation and PA as children approach adolescence, and that boys are more likely to participate in sports and engage in more PA than girls [33–35]. Higher family incomes have predicted youth sport participation and PA in younger age groups (6–9 year olds) [36]. A 2015 study of U.S. households found that material resources mattered. Families with more income had significantly higher levels of children participating in organized activities [37]. Children in rural settings may have less access to youth sport because of lack of programming and transportation barriers [6].

There is a gap in our understanding of the factors related to youth sport participation in rural settings and how these factors interact to influence PA outcomes [14, 38]. The purpose of this study was to examine the influence of grade, sex, and family income on youth sport participation and these factors and youth sport participation on moderate-to-vigorous physical activity of children in rural communities.

## Methods

### Study sample

This study was a sub-study of the Wellscapes Project. The ongoing Wellscapes Project is both a Type 3 Hybrid-Implementation-Effectiveness community randomized trial (ClinicalTrials.gov Identifier: NCT03380143) of four rural communities and a social epidemiology study to determine the impact of system drivers on child population physical activity [39, 40]. In Wave 1 of the Wellscapes Project, two rural Great Plains communities representing communities with a majority of primarily white children in 3rd through 6th grade were targeted for planned recruitment. In Wave 2 of the Wellscapes Project, two communities representing communities with a majority of primarily Hispanic/Latino children in 3rd through 6th grade will be targeted for planned recruitment [39]. Inclusion criteria for the Wave 1 communities (*n* = 2) were the following: concentration of white, non-Hispanic residents; completion of a community health needs assessment and prioritization of obesity prevention on a community health improvement plan; location in a rural area that was distant from an urbanized area (> 10 miles), had one public high school; and agreement by the health department and school district to participate in the study.

The present study reports on Spring of 2019, 3rd–6th graders (*n* = 418) participating in the Wave 1 social epidemiology component who completed the PA surveillance instrument. This study also reports on a subset of children (*n* = 235) participating in the two communities’ targeted classrooms, after school programs, youth clubs and youth sport who were recruited to be a part of the Wellscapes Project community trial and were included in the trial upon receipt of informed parental consent. Children who participated in the community trial and also completed the PA surveillance instrument, had parental consent to link their school demographic data (i.e., sex, grade, and free and reduced lunch status) with their surveillance instrument responses. The Institutional Review Board at University of Nebraska Medical Center approved data collection procedures in 2018 (IRB #446–18-EP).

### Measures

#### Demographics

Covariates in this study included grade, sex, and family income. Grade was either 3rd, 4th, 5th, or 6th grade, based on each student’s school enrollment records during Spring 2019, and sex was either male or female. Family income was based on family free and reduced lunch status. We created a dichotomous variable with lower income based on either free or reduced lunch status and higher income based on full-pay lunch status.

#### Youth sport participation

Youth sport participation was obtained using a set of supplemental survey items in the Youth Activity Profile that are based on items from the National Survey of Children’s Health 2017–2018 (NSCH) [32]. These items assessed participation in out-of-school activities including daily afterschool programs, sports teams or sports lessons, clubs or organizations (i.e., 4-H, Scouting), and other organized activities or lessons (i.e., music, dance, language, arts). The present analyses relied on “yes” or “no” responses to the following question pertaining to the out-of-school activity of youth sport: “During the past 12 months, did you participate in a sports team or take sports lessons after school or on weekends?” [32].

#### Moderate to vigorous physical activity

The primary outcome variable of children’s MVPA was obtained from the Youth Activity Profile (YAP), a validated online, self-report assessment of children’s PA behaviors [41]. The YAP comprises 15 items that assess time-segmented youth PA, both in-school (5 items) and out-of-school (5 items), as well as sedentary behavior (5 items). Consistent with standardized protocols, students completed the YAP during the school day with teacher supervision and were reminded to reflect specifically on their behaviors in the last week. Individualized reports from the YAP platform were provided to individual students, but a novel aspect of the YAP is that the data can be aggregated to provide accurate group level estimates of child PA and sedentary behavior [41]. The estimates were obtained using updated calibration equations developed specifically for the online version of the YAP (Welk, unpublished observations). The equations had computed values of Mean Absolute Percent Error (MAPE) ranging from 15 to 25% when compared to monitor-based estimates and have suitable precision for the present analyses.

### Analyses

Descriptive statistics were examined to summarize the social epidemiology study participation and the clinical trial participation samples and the measures. Mixed-models were used to analyze the continuous PA outcome of daily average minutes of MVPA (in-school and out-of-school) and the dichotomous outcome of youth sport participation using MIXED and GLIMMIX procedures respectively, from SAS/STAT software, version 9.4 (© 2002–2012) of the SAS System for Windows (Cary, NC). For all models, community was included as a random effect. Grade, sex, and family income and their interactions were used as fixed effects, and model analyses were conducted on generated least squares means. The PA outcome model also included youth sport participation as a fixed effect. To assure that over-fitting was not a problem, backward elimination of non-significant covariates based on statistical significance of *p* < 0.05 was used where non-significant higher order interactions were eliminated first, and then the models were refit [42, 43].

## Results

### Demographics

Descriptive characteristics of children in the Wellscapes YAP social epidemiology study along with characteristics of the subset of children consented to participate in the Wellscapes community trial can be found in Table 1. A total of 418 3rd–6th graders participated in Spring of 2019 across two communities. Of those, 235 students consented to participate in the community trial. In the community trial, more children participated in youth sport than did not (80% versus 20%), and more children had a full-pay lunch status than free/reduced lunch status (75.3% versus 24.7%).

**Table 1:** Child Characteristics

	Social Epidemiology Study Participation	Clinical Trial Participation
School 3rd – 6th	*n* (%)	*n* (%)
3rd – 6th	418	235
3rd	108 (25.8)	56 (23.8)
4th	100 (23.9)	56 (23.8)
5th	117 (28.0)	61 (26.0)
6th	93 (22.2)	62 (26.4)
Sex		
Female	209 (50.0)	118 (50.2)
Male	209 (50.0)	117 (49.8)
Youth Sport Participation		
Yes	302 (72.2)	188 (80.0)
No	116 (27.8)	47 (20.0)
Lunch Status		
Free/Reduced		58 (24.7)
Full Pay		177 (75.3)

### Youth sport participation

Table 2 shows the results from the mixed-model regression predicting youth sport participation. Full-pay lunch status students were almost four times more likely to participate in youth sport than students with a free or reduced lunch status (OR = 3.91, 95% CI = 1.95, 7.8). There were no significant differences by grade level or sex and the odds of participating in youth sport.

**Table 2:** Odds ratios predicting youth sport participation

Child characteristics	Odds Ratio (95% CI)
Grade	
3rd	reference
4th	1.91 (0.65–5.6)
5th	0.77 (0.31–1.9)
6th	1.10 (0.42–2.8)
Sex	
Male	reference
Female	.73 (0.37–1.5)
Lunch status	
Free/reduced	reference
Full pay	3.91 (1.95–7.8)*

*p < 0.05

### Moderate to vigorous physical activity

Table 3 presents least squares means and significant differences in daily MVPA by main effects of grade, sex, lunch status, and youth sport participation. There were significant differences in MVPA by grade, with a drop in average minutes of MVPA among 6th graders, as compared to 3rd, 4th, or 5th graders (74.6 min compared to 83.9, 80.5, and 87.0 min, respectively). Males reported significantly more minutes of MVPA than females (93.7 min versus 69.3 min). There were no significant differences in MVPA by lunch status, but there were significant interaction effects on MVPA between lunch status and sex and lunch status and grade. Table 4 presents least squares means estimates and significant differences in daily MVPA by these interaction effects. Males with a free/reduced lunch status had approximately 4.8 min fewer daily minutes of MVPA than those with a full-pay status. In contrast, females with free/reduced lunch status had approximately 4 min more of MVPA than females with a full-pay status. There were also significant interaction effects of grade and lunch status for all grades. 6th graders with a full-pay lunch status reported significantly fewer minutes of daily MVPA as compared to those with free/reduced lunch status (71.2 min vs. 78.0), and there was a significant difference in minutes of daily MVPA between 3rd and 6th graders with a full-pay lunch status, dropping over 13 min from 84.4 min to 71.2.

**Table 3:** Least squares means estimates of total minutes of daily MVPA by child characteristics

MVPA, adjusted mean (95% CI)		
Characteristic		Differences^a1^ (*p* < 0.05)
Grade		
a. 3rd	83.9 (51.6–116.2)	d
b. 4th	80.5 (49.7–111.3)	c,d
c. 5th	87.0 (48.1–126.0)	b,d
d. 6th	74.6 (38.4–110.8)	a,b,c
Characteristic		Differences^b2^ (*p* < 0.05)
Sex		
a. Female	69.3 (25.2–113.4)	b
b. Male	93.7 (52.0–135.4)	a
Characteristic		Differences^c3^ (*p* < 0.05)
Lunch Status		
a. Free/Reduced	81.3 (42.7–120.0)	None
b. Full Pay	81.7 (34.6–128.8)	None
Characteristic		Differences^d4^ (*p* < 0.05)
Youth Sport Participation		
a. Yes	84.9 (36.0–113.8)	b
b. No	78.1 (41.7–114.4)	a

*MVPA* moderate to vigorous physical activity, *CI* confidence interval

^a1^ Significance from mixed effects model (e.g., ‘a’ denotes difference from 3rd grade)

^b2^ Significance from mixed effects model (e.g., ‘a’ denotes difference from female)

^c3^ Significance from mixed effects model (no significant differences found)

^d4^ Significance from mixed effects model (e.g., ‘a’ denotes difference from yes to youth sport participation)

**Table 4:** Least squares means estimates of total minutes of daily MVPA by lunch status interactions with sex and grade

	MVPA mean (95% CI)	Differences^a1^ (*p* < 0.05)
Free/Reduced Lunch Status		
a. Male	91.3 (63.7–119.0)	b,c,d
b. Female	71.3 (40.2–102.3)	a,c,
Full-Pay Lunch Status		
c. Male	96.1 (54.6–137.6)	a,b,d
d. Female	67.3 (25.6–109.0)	a,c
	**MVPA mean (95% CI)**	**Differences**^**b2**^ (***p* < 0.05)**
Free/Reduced Lunch Status		
e. 3rd grade	83.4 (63.8–103.0)	h
f. 4th grade	76.2 (57.3–95.1)	c,e,f,g
g. 5th grade	87.6 (61.2–113.9)	b,d,h
h. 6th grade	78.0 (55.8–100.3)	c,e,f,g,h
Full-Pay Lunch Status		
i. 3rd grade	84.4 (49.6–119.1)	b,d,h
j. 4th grade	84.7 (50.8–118.6)	b,d,h
k. 5th grade	86.5 (53.1–120.0)	b,d,h
l. 6th grade	71.2 (36.8–105.5)	a,c,d,e,f,g

*MVPA* moderate-to-vigorous physical activity, *CI* confidence interval

^a1^ Significance from mixed effects model (e.g., ‘a’ denotes difference from male)

^b2^ Significance from mixed effects model (e.g., ‘a’ denotes difference from 3rd grade)

**Fig. 1:**
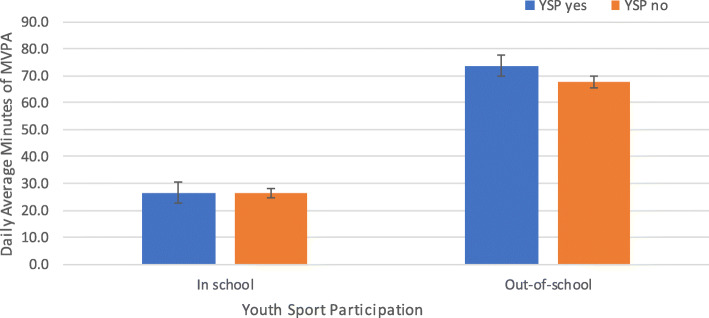
Differences between youth sport participation and in-school and out-of-school daily average minutes of MVPA

There were significantly more daily average minutes of MVPA for those children that participated in sports than those who did not participate (84.9 min vs. 78.1, *p* < 0.05). Figure 1 shows differences between youth sport participation and in-school and out-of-school daily minutes of MVPA. In-school MVPA was similar for those with youth sport participation and those without youth sport participation (26.6 and 26.3 min, *p* = 0.76), but out-of-school daily MVPA differed by youth sport participation by 6 min between the groups (73.8 min vs. 67.8, *p* < 0.05). We found even greater differences in out-of-school MVPA for those children that participated in sports than those that did not participate when analyzing the full social epidemiology study sample (73.2 min vs. 63.1, *p* < 0.05).

## Discussion

This study examined the influence of grade, sex, and family income on youth sport participation and their impact on MVPA of children in rural communities. While there were no significant impacts of grade on youth sport participation, there were significant impacts of grade on PA—with the level of average daily minutes of MVPA lowering by grade with the sharpest drop of over 12 min per day from 5th grade to 6th grade. In a 2007 review of worldwide youth sport trends, there was a steady decline in youth sport participation in 11 European countries starting during adolescence through to adulthood [33]. Reasons for dropping out included lack of time, less interest, less perceived competence, less support from family and friends, and participation in other leisure activities [33]. According to the Aspen Institute/Utah State University 2019 National Youth Sport Survey, most children in the U.S. quit playing sports by the age of 11 [44]. Declines in youth sport participation as children age in the U.S. may be explained by the competitive and exclusionary nature of both school and club sports that often starts around adolescence [44]. Our study does not show a significant decline in youth sport participation by grade among rural 3rd-6th graders, but a lower level of MVPA, which may indicate that time actually being physically active is lacking in in-school or out-of-school settings that include youth sport (e.g., recess, physical education, out-of-school clubs). A study of the contribution of youth sport to PA among boys aged 6–12 years found that youth sport contributed to 23% of their daily MVPA [25], and more recent research found that participating in sports doubled the odds of meeting PA guidelines in similar aged children [29]. It appears youth sport participation is making a positive impact on daily MVPA. It may be the quantity of active minutes of PA during youth sport is declining as children get older, which is an important question for further research. Similar to our findings, a 2005 review of evidence for trends in children’s PA behaviors found a worldwide decline in children’s aerobic performance between 1995 and 2005 [13]. A 2019 study found a decline in children’s cardiorespiratory fitness in high and upper middle-income countries between 1981 and 2014 [45]. Studies that examine the quantity and quality of children’s PA during youth sport in a variety of settings, in both urban and rural communities, may shed light on the contextual factors that explain declines in MVPA as children age.

Sex was not significantly associated with youth sport participation, but had a significant impact on MVPA, with males in our study averaging almost 25 more minutes of physical activity per day. This finding is consistent with past self-report and objective measure studies showing variations in PA by sex, with boys engaging in more MVPA than girls [1, 34, 35]. We did, however, find an interaction between sex and family income on MVPA. Males with a full-pay lunch status were significantly more active than males with a free/reduced status. Our findings show that the opposite was true for females. Free/reduced lunch status females were more active than full-pay lunch status females. Interestingly, we do not see an interaction effect between sex, lunch status, and youth sport participation for males or females, so youth sport participation may not be the opportunity that explains these interactions. It may be that where children spend out-of-school time varies by sex and socioeconomic status. But we can safely say that youth sport does not appear to be a driver of differences. This is a question for further research. Nonetheless, given the significantly lower levels of MVPA reported by girls in our study, targeted interventions may be needed across in-school and out-of-school settings that help to increase PA among girls. Past research has shown that girls’ participation in sport declines after adolescence [33], and our study included girls only through the 6th grade. Youth sport programs that aim to include girls post-adolescence and that are tailored to keep them motivated to participate may have an impact on girls’ MVPA over time.

We found that family income had a significant effect on youth sport participation, with higher incomes predicting almost four times higher youth sport participation. This finding aligns with prior research on socioeconomic status and participation [12, 36, 37]. Though this effect in rural settings may not be as apparent as previous research suggests, with 80 % of the children in our study reporting participating in youth sport within the past year [6]. Family income did not have a significant impact on MVPA, but those children who participated in sports had significantly greater MVPA, by approximately 7 min more per day. Perhaps, therefore, the impact of family income on MVPA is mediated by youth sport participation. The impact of family income on MVPA was also dependent on grade. Unexpectedly, we observed a drop in levels of MVPA by grade that was more apparent with students that were full-pay lunch status than those that were free/reduced lunch status. Perhaps a decline in PA by age and income is less realized in rural communities where, especially in elementary school, there is a need for teams to fill their rosters and therefore be more inclusive. Barker posits in his behavior setting theory that in smaller towns there is a social demand for sustained participation in a variety of activities and settings due to a short supply for positions to be filled [46, 47]. Research also shows a short supply, or setting demand, on a sports team may mean more play time, physical activity, and satisfaction for those participating [48–50]. Reducing costs and barriers to participate in youth sport, though, could enable children from all income levels to be more physically active as they move out of elementary school into junior high and high school when distance and transportation become factors [38].

Past strategies for improving children’s PA behaviors have focused on schools as the lead coordinating institution for implementation, but more recently the 2013 Physical Activity Guidelines Midcourse Report called for a focus on other settings [14, 51]. We found very little difference in in- school MVPA between those who participated in youth sport and those that did not, but we found that children participating in youth sport had 6 min more per day of out-of-school MVPA. This finding suggests that schools may not be the only setting in rural communities where we should focus our attention. Rural communities may see significantly more impact on children’s PA with improved youth sport delivery implemented through community collaborations. Other possible coordinating institutions within rural communities include parks and recreation agencies and libraries and including stakeholders from these organizations in collaborations may increase local capacity for youth sport interventions.

### Limitations

This study is not without limitations. Our sample was limited to two communities, but these communities were selected as representative of rural Great Plains communities that lack racial and ethnic diversity. The next wave of the Wellscapes Project will recruit representative rural Great Plains communities with a concentration of Hispanic-Latino children. Future research should investigate the impact of race and ethnicity on youth sport participation and other opportunities to be active and MVPA within a rural context, as these are important considerations in assessing the accessibility of programs and their impact on health outcomes. Child PA was self-reported and may not as accurately reflect MVPA as well as objective measures like accelerometry and observation, though the YAP is a tool that has been extensively validated and captures self-report data as accurately as possible. In fact, group level estimates from the YAP in a preliminary calibration study were statistically equal to the SenseWear Armband Pro3 PA monitor [41].

## Conclusions

This study suggests that, along with other factors, youth sport participation plays an important role in the daily PA of children in rural communities. While a fairly high percentage of children have youth sport participation, there are disparities in rural communities on youth sport participation and PA outcomes based on age, sex, and family income. Because youth sport is a popular out-of-school setting for PA in rural communities, more accessible youth sport programming could make a significant impact on the MVPA and health of children.

## Data Availability

Following NIH and IRB data sharing policies, data will be made available after publication of the main findings of R01CA215420 from the PI David A. Dzewaltowski, Ph.D.

## Abbreviations

PA: Physical activity
MVPA: Moderate to vigorous physical activity
NSCH: National Survey of Children’s Health
YAP: Youth Activity Profile
MAPE: Mean Absolute Percent Error
